# Antibodies against a β-glucan-protein complex of *Candida albicans* and its potential as indicator of protective immunity in candidemic patients

**DOI:** 10.1038/s41598-017-02977-6

**Published:** 2017-06-02

**Authors:** Antonella Torosantucci, Mario Tumbarello, Carla Bromuro, Paola Chiani, Brunella Posteraro, Maurizio Sanguinetti, Roberto Cauda, Antonio Cassone

**Affiliations:** 1 Department of Infectious, Parasitic and Immune-mediated Diseases, Istituto Superiore di Sanità, 00161 Rome, Italy; 2 Institute of Infectious Diseases, Catholic University of the Sacred Heart, 00168 Rome, Italy; 3 Institute of Public Health, Catholic University of the Sacred Heart, 00168 Rome, Italy; 4 Institute of Microbiology, Catholic University of the Sacred Heart, 00168 Rome, Italy; 5 Polo d’innovazione della genomica, genetica e biologia, University of Perugia, 71000 Perugia, Italy

## Abstract

Sera from candidemic and non-candidemic subjects were examined for antibodies against the cell wall β1,3- and β1,6-glucans, as well as the β-glucan-associated protein MP65 of *Candida* species. Although antibodies against each of the above components were detected in all subjects, candidemic patients had lower antibody titers against β1,3-glucan, but higher antibody titers against β1,6-glucan and MP65, than non-candidemic subjects. The elevated levels of anti-β1,6-glucan and -MP65 antibodies found in candidemic patients were independent on the patient risk category, APACHE II score, presence of co-morbidities, β1,3-glucanemia level, *Candida* isolate, and antifungal treatment. Interestingly, however, the anti-MP65, but not the anti-β1,6-glucan antibodies, of candidemic patients had higher titers in survivors than in non-survivors, particularly in those subject categories with the highest mortality (>65-years old, diabetic, or septic shock patients). Thus, candidemic patients are capable of boosting anti-*Candida* immune responses upon infection, and some of these responses might be associated to the generation of protective immunity in patients with candidemia.

## Introduction

Candidemia is a remarkable health threat to hospitalized patients, including non-neutropenic subjects with advanced age and co-morbidities^1–3^. Despite progress in epidemiology, diagnosis and therapy, candidemia remains associated with high mortality (30–50%) and increasing incidence^1, 3–7^. In some hospital settings, *Candida* spp. are among the most frequent causes of bloodstream infections^1, 8, 9^.

While many risk factors which make a subject prone to develop the disease are known, and include deep surgery and a number of invasive, medical-associated technologies, those influencing disease outcome are only partially known and remain somewhat debated^10^. Data on causative *Candida* spp. and their antifungal resistance are widely available and suggest influence of local epidemiology on infection and possibly also on its outcome^8, 11–14^. A role for older age, inappropriate or non-timely antifungal therapy and general patient conditions on mortality rate appears to be plausible, though marked differences among the different studies have been reported^5, 8, 13–16^.

Only few studies have directly addressed the presence and magnitude of anti-*Candida* immunity, in particular antibody responses, in the above patients, mostly with the aim of providing diagnostic indicators^17–24^. We are studying antibody responses in candidemia with the main purpose of assessing whether and to what extent antibodies to key structural and/or functional fungal antigens could be used as prognostic indicators and as a guide to identify new vaccine candidates. Following this research line, we have now focused on antibodies against a major polysaccharide-protein complex that is commonly present in *Candida* species causing candidemia. This complex contains both β1,3- and β1,6 glucans, of which the former is a currently accepted diagnostic marker of candidemia and a molecular pattern recognized by a major receptor, Dectin-1, commonly expressed on cells of innate immunity^25, 26^. The complex also contains a β-glucan-associated mannoprotein, MP65, with a putative endo-glucanase activity which plays a critical role for cell wall growth and stability, and is a major target of anti-*Candida* adaptive immunity in humans^27, 28^.

Here we report that candidemic subjects are able to boost the anti-*Candida* response normally associated with colonization, and suggest that the capacity of boosting some of the above responses could indicate the generation of protective immunity, hence impact on disease outcome.

## Results

### Clinical and laboratory data

Seventy one candidemic patients (40 males and 31 females; mean age 67.2 ± 3.1; median 72.0) and sixty nine age- and sex-matched control patients entered this study. All candidemic patients had *Candida*-positive blood cultures, positive β-glucanemia and signs and symptoms of the infection. For candidemic patients, the median duration of hospitalization and the APACHE II score at the time of the first positive blood culture were 21.5 days (range 2–180) and 16 (range 5–37), respectively. Thirty our subjects had a central venous catheter inserted. About half of the patients underwent previous surgery and for only seven of them there was evidence for immunosuppression. The vast majority of patients were treated with anidulafungin or caspofungin.

The controls were patients who were admitted to the hospital within the same period of time as the candidemic ones and were considered at risk for candidemia, but with *Candida*-negative blood-culture and negative β-glucanemia. Among them, 25 had a central venous catheter inserted, 30 had previous surgery, 6 were diabetics and 39 had previous antibiotic treatment. Other details are reported in the Supplementary Table 1.

Isolates from six *Candida* species were obtained from candidemic subjects, about 60% being *C*. *albicans*, alone or together with another species, followed by *C*. *parapsilosis* (19.7%), *C*. *glabrata* (7.0%), *C*. *tropicalis* (7.0%), and one isolate each of other minor *Candida* species. Of our patients, 31 (43.7%) did not survive. The mortality appeared to be associated with older age (P = 0.03), septic shock (P = 0.008), and diabetes mellitus (P = 0.008) but neither with APACHE II score, previous surgery, or other co-morbidities. The clinical data are detailed in Table 1.

**Table 1:** Demographic, clinical and microbiological data of candidemic patients considered in the study.

	All patients	Survivors	Non-survivors	P, survivors *vs* non-survivors	Odds ratio (95% confidence interval)
**Candidemic patients, n (%)**	71	40 (56.3)	31 (43.7)		
**Sex**					
Male, n/total (%)	40/71 (56.3)	22/40 (55.0)	18/40 (45.0)	0.81^a^	0.88 (0.34–2.27)
Female, n/total (%)	31/71 (43.7)	18/31 (58.1)	13/31 (41.9)		
Age, median (range)	72 (14–91)	67.5 (20–86)	76 (14–91)	**0.03** ^b^	
**Isolated** ***Candida*** **species, n/total (%)**					
*C*.*albicans*	38/71 (53.5)	21/40 (52.5)	17/31 (54.8)		
*C*.*parapsilosis*	14/71 (19.7)	11/40 (27.5)	3/31 (9.7)		
*C*.*glabrata*	5/71 (7.0)	2/40 (5.0)	3/31 (9.7)		
*C*.*tropicalis*	5/71 (7.0)	2/40 (5.0)	3/31 (9.7)	1.0^c^	0.91 (0.35–2.33)
*C*.*krusei*	1/71 (1.4)	1/40 (2.5)	0/31 (0)		
*C*.*guilliermondii*	1/71 (1.4)	0/40 (0)	1/31 (3.2)		
*C*.*lusitaniae*	1/71 (1.4)	1/40 (2.5)	0/31 (0)		
*C*.*robusta*	1/71 (1.4)	0/40 (0)	1/31 (3.2)		
*C*.*albicans* associated with other *Candida* species	5/71 (7.0)	2/40 (5.0)*	3/31 (9.7)**		
**Antifungal treatment** ^**d**^					
Anidulafungin	34/71 (47.9)	16/40 (40.0)	18/31 (58.0)		
Caspofungin	19/71 (26.8)	12/40 (30.0)	7/31 (22.6)	0.59^e^	
Fluconazole	19/71 (26.8)	11/40 (27.5)	8/31 (25.8)		
Liposomal Amphotericin B	9/71 (12.7)	6/40 (15.0)	3/31 (9.7)		
Serum beta-glucan at the time of the first positive blood culture, median pg/ml (range)	>500 (97->500)	>500 (97->500)	>500 (135->500)	0.66^b^	
Total duration of hospital stay at the time of the first positive blood culture, median days (range)	21.5 (2–180)	20 (2–71)	23 (3–180)	0.58^b^	
Median (range) APACHE II score at the time of the fist positive blood culture	16 (5–37)	15 (5–35)	16 (5–37)	0.26^b^	
Septic shock, n/total (%)	37/71 (52.1)	15/40 (37.5)	22/31 (80.0)	**0.008** ^a^	4.07 (1.49–11.14)
Central venous catheter, n/total (%)	34/71 (47.8)	18/40 (45.0)	16/31 (51.6)	0.64^a^	1.30 (0.50–3.33)
Previous surgery, n/total (%)	34/71 (47.8)	18/40 (45.0)	16/31 (51.6)	0.64^a^	1.30 (0.50–3.33)
Hematomalignancy, n/total (%)	5/71 (7.0)	2/40 (5.0)	3/31 (9.7)	0.65^a^	2.03 (0.32–13.01)
HIV, n/total (%)	1/71 (1.4)	0/40 (0)	1/31 (3.2)	na	na
Immunosuppressant, n/total (%)	7/71 (9.8)	5/40 (12.5)	2/31 (6.5)	0.46^a^	0.48 (0.09–2.68)
Steroids, n/total (%)	20/71 (28.1)	13/40 (32.5)	7/31 (22.6)	0.43^a^	0.61 (0.21–1.77)
Solid tumor, n/total (%)	16/71 (22.5)	8/40 (20.0)	8/31 (25.8)	0.58^a^	1.39 (0.46–4.25)
Chronic obstructive pulmonary disease, n/total (%)	20/71 (28.2)	11/40 (27.5)	9/31 (29.0)	1.00^a^	1.08 (0.38–3.05)
Chronic renal failure, n/total (%)	13/71 (18.3)	8/40 (20.0)	5/31 (16.1)	0.76^a^	0.77 (0.22–2.64)
Diabetes mellitus, n/total (%)	16/71 (22.5)	4/40 (10.0)	12/31 (38.7)	**0.008** ^a^	5.68 (1.61–20.06)
Previous antibiotic treatment (30 days), n/total (%)	54/71 (76.1)	32/40 (80.0)	22/31 (70.9)	0.41^a^	0.61 (0.20–1.83)

^a^Fisher exact test; ^b^Mann-Whitney U test;

*C.*albicans + C*.*krusei* (1), *C*.*albicans + C*.*guilliermondii* (1); **C.*albicans + *C.*glabrata*

^c^Fisher exact test evaluating isolation of *non-albicans* species, alone or in association, *vs* the isolation of

*C*.*albicans* only

^d^Nine patients were treated with more than one antifungal

^e^Chi-square test

na = not applicable.

### Levels of serum anti-β-glucan or anti-MP65 IgG antibodies in candidemic patients

**Figure 1:**
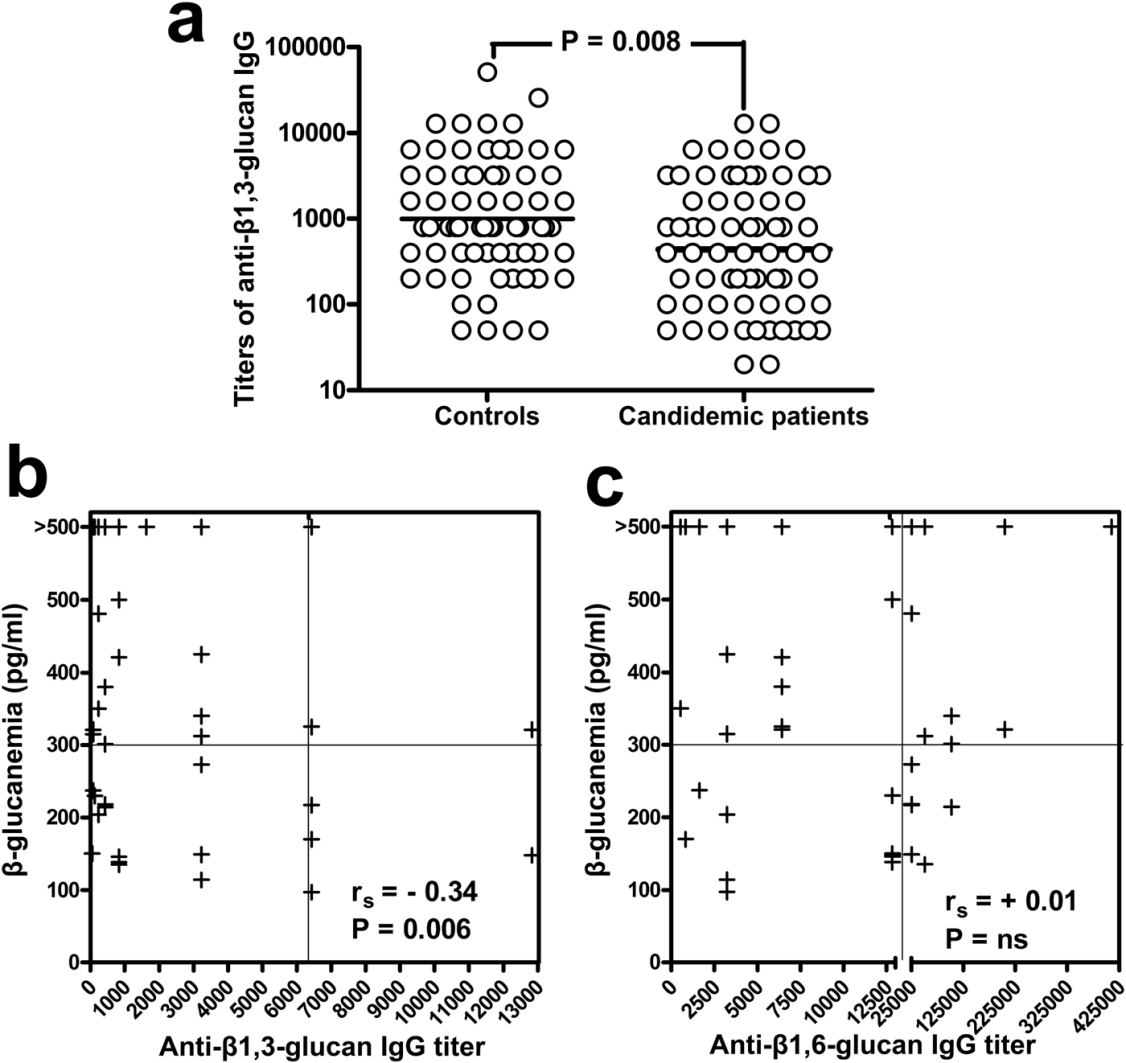
Serum titers of anti-β1,3-glucan IgG are lower in candidemic patients than in controls, and are inversely correlated to levels of circulating β1,3-glucan. Panel a: Individual, anti-β1,3-glucan IgG titers in patients with candidemia (n = 71) and in non-candidemic controls (n = 69). A single serum samples was analyzed for each subject. For candidemic patients, sera were those obtained at onset of candidemia, i.e. from the same blood specimen of the first *Candida*-positive blood culture. Geometric means are indicated by the lines. P was estimated by the Mann-Whitney U test. Panels b and c: Correlation between serum titers of anti-β1,3-glucan (**b**) or anti-β1,6-glucan (**c**) IgG and beta-glucanemia in candidemic patients. Correlation coefficients r_s_ and P values were calculated by the Spearman’s rank correlation analysis.

Presence and titers of anti-β-glucan and anti-MP65 antibodies at onset of candidemia were determined in sera from candidemic patients, taken at time of laboratory diagnosis of the first candidemia episode, and compared to those of non-candidemic controls. Both laminarin (β1,3-glucan) and pustulan (β1,6-glucan) were initially used as representative of *Candida* β-glucan antigens. However, most candidemic patients showed high levels (≥500 pg/ml; Table 1) of circulating β1,3-glucan that could strongly bind the respective specific antibodies and make them poorly detectable in our ELISA test. In support of this interpretation, we found that our candidemic patients had significantly lower titers of anti-β1,3-glucan (laminarin) antibodies, as compared to the control group (Fig. 1A), and that their levels of anti-β1,3-glucan IgGs were inversely related to the amount of serum β1,3-glucan (Fig. 1B). In contrast, there was no significant correlation between β1,3-glucanemia and titers of anti-β1,6-glucan (pustulan) IgGs (Fig. 1C). For this reason, only the anti-β1,6-glucan IgGs were further examined in this study as representative of genuine titers of antibody against *Candida* β-glucan.

**Figure 2:**
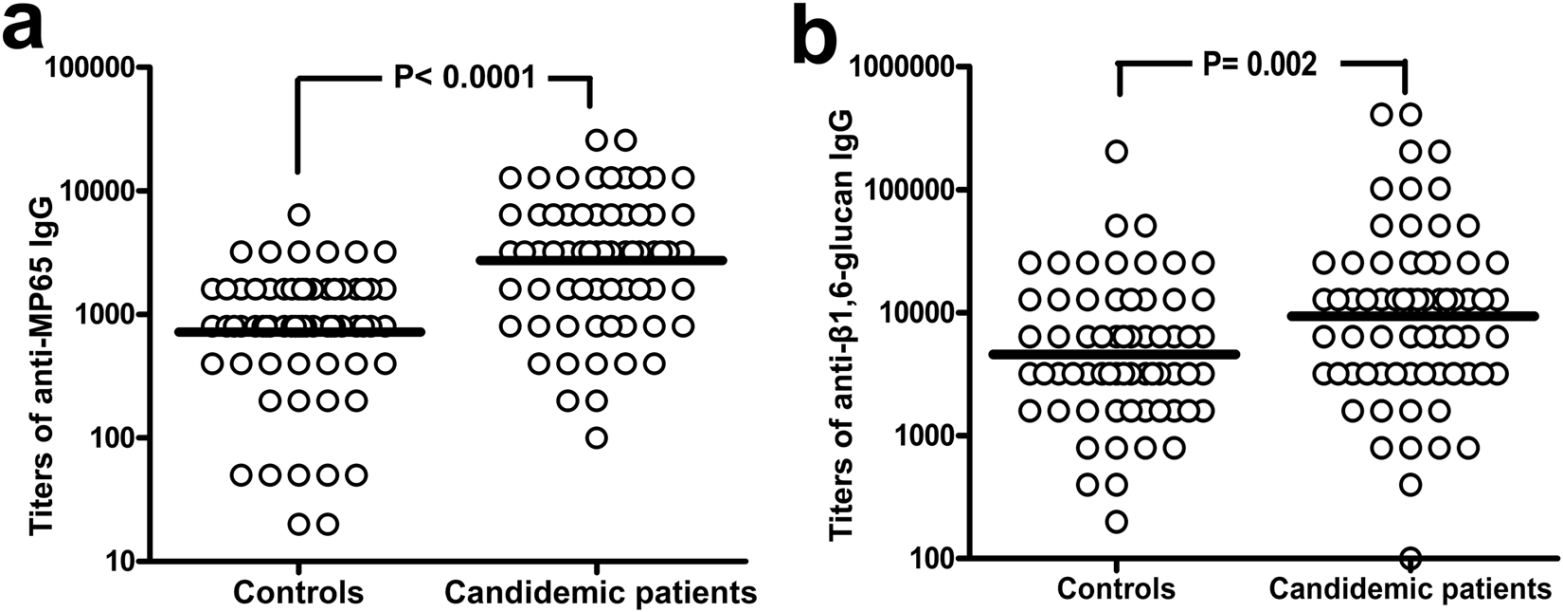
Onset of candidemia is associated to a significant rise of anti-MP65 and anti-β1,6-glucan antibodies. The graph shows the individual titers of anti-MP65 (Panel a) or anti-β1,6-glucan IgGs (Panel b) measured in control (n = 69) and candidemic patients (n = 71). Sera of candidemic subjects (one for patient) were from the same blood specimen of the first *Candida*-positive blood culture. Lines are at geometrical mean titers. P values were calculated by the Mann-Whitney U test.

Figure 2 shows IgG titers against β1,6-glucan and MP65 in candidemic patients in comparison with the same antibodies in controls. The candidemic patients had higher IgG titers than controls against both MP65 (P < 0.0001) and β1,6-glucan (P = 0.002). The ROC curves generated from these assays (Supplemental Figure S1) showed that titers of anti-MP65 IgGs could better discriminate candidemic from non-candidemic patients (AUC = 0.78, sensitivity 76 or 62% and specificity 59 or 90% at a cutoff of > 1200 or >2400, respectively) than those of anti-β1,6-glucan IgGs (AUC = 0.65, sensitivity 55 or 40% and specificity 52 or 40% at a cutoff of >4800 or >9600, respectively).

The specificity of our anti-MP65 IgG ELISA was confirmed by qualitative western blot analyses of representative sera from candidemic and non-candidemic subjects using an anti-MP65 monoclonal antibody as positive control^27^. These assays clearly showed that the binding pattern of human sera substantially overlapped the one exhibited by the MP65-specific monoclonal antibody, with a reaction intensity in good apparent correlation with the anti-MP65 ELISA titers (Supplemental Figure S2).

**Figure 3:**
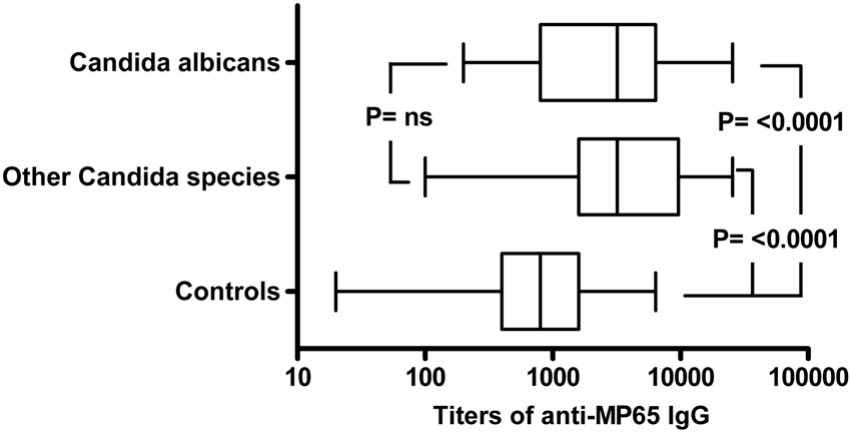
Levels of anti-MP65 IgGs are comparably increased in patients infected by *C*. *albicans* or by other, non-*albicans*, *Candida* species. The plot compares the distribution of anti-MP65 IgG titers in patients with *C*. *albicans* candidemia (n = 43), patients infected by other *Candida* species (n = 28) and non-candidemic controls (n = 69). Bottom and top of the box represent the first and third quartiles, respectively. Line inside the box is the median titer and whisker show minimum and maximum values. P was calculated by Kruskal-Wallis ANOVA and Dunn’s post-test.

The difference in the levels of anti-MP65 serum antibodies between candidemic and non-candidemic patients was significant both in patients infected by *C*. *albicans* (P < 0.0001 *vs* controls) and in those infected by non*-albicans Candida* species (P < 0.0001 *vs* controls, Fig. 3). The anti-MP65 IgG titers were overall similar (P > 0.05) in candidemic patients receiving or not receiving supportive steroids and with or without septic shock. However, the titers of anti-MP65 IgGs in diabetic patients with candidemia had the lowest levels over those in control population (geometric mean 1,467, 95% CI 685–3141 *vs* 726, 541–975; P = 0.37) compared to the titers in non-diabetic, candidemic patients (geometric mean 3,160, 95% CI 2,239–4,269; P < 0.0001). There was no statistically significant correlation between β1,3-glucanemia values and anti-MP65 IgG titers, nor there was correlation between antibody levels and patient age or APACHE II score (data not shown).

### Relationship between levels of anti-MP65 IgGs and survival of candidemic patients

**Figure 4:**
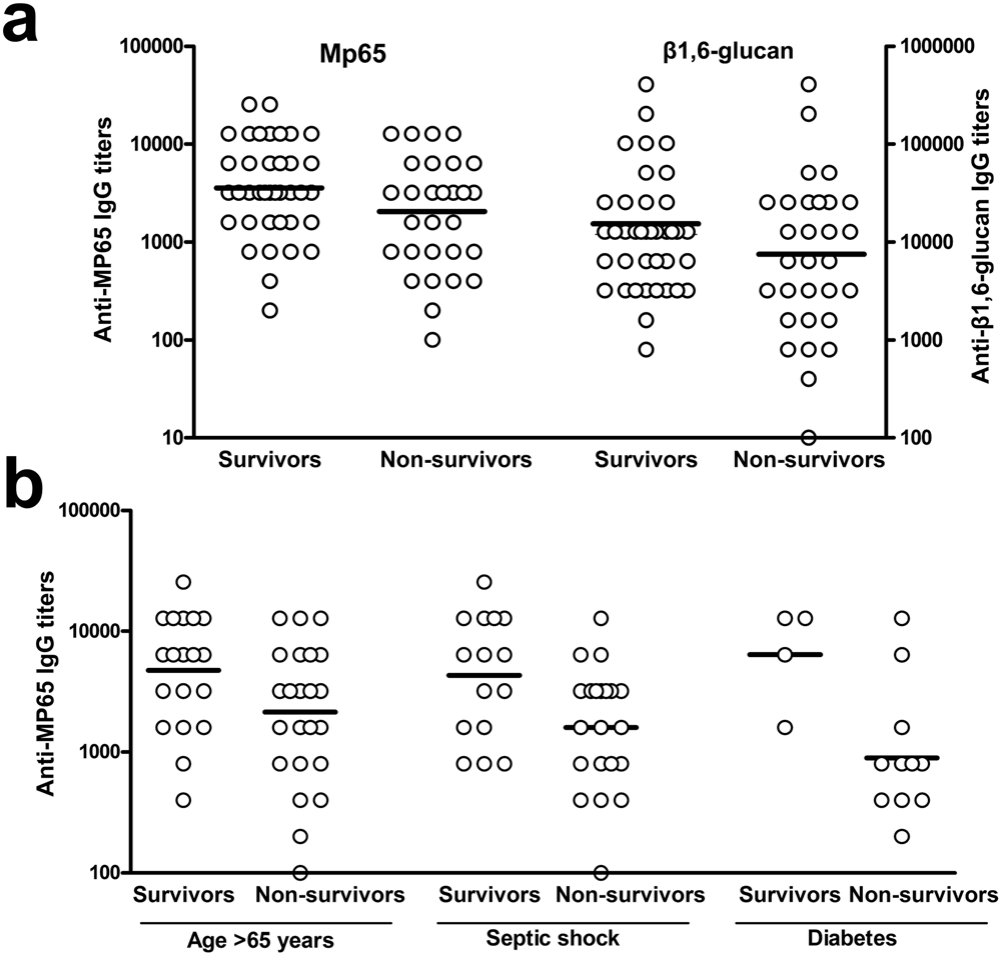
Comparison of anti-MP65 and anti-β1,6-glucan antibody levels at candidemia diagnosis in survivors and non-survivors among candidemic patients. Panel a: Distribution of individual IgG titers against MP65 or β1,6 glucan in survivors (n = 40) and non-survivors (n = 31). Panel b: Levels of anti-MP65 IgGs in surviving and non-surviving candidemic patients belonging to categories of high mortality risk in our setting (patients aged >65 years, with septic shock or with diabetes mellitus, n survivors vs n non-survivors = 22 vs 25, 15 vs 22 and 4 vs 12, respectively) Logistic regression analysis was used to calculate the Odds ratio for the relationship between survival and anti-MP65 and anti–β1,6-glucan antibody titers.

We examined whether there was any association between survival of patients with candidemia and titers of anti-β1,6-glucan or anti-MP65 antibodies at candidemia diagnosis. As show in Fig. 4A, the survivors had overall higher antibody titers against both β1,6-glucan and MP65 as compared to the non-survivors. In the case of MP65 antigen, the higher titers of survivors were statistically significant, a finding particularly notable in patients with the highest mortality in our setting, as those of older age, or diabetic or with septic shock (Fig. 4B). In fact, logistic regression analysis showed a significant, positive relationship between values (logarithm) of anti-MP65 titer and probability of survival, both in the whole population of candidemic patients (OR 2.61, Cl 95% 1.01–6.75, p = 0.047) and in all three, high mortality-risk subpopulations (OR 17.7, Cl95% 1.10–284.8, p = 0.041 in diabetic patients; OR 4.38, Cl 95% 1.01–19.04, p = 0.048 in patients with septic shock; OR 3.78, Cl 95% 1.07–13.29, p = 0.038 in patients of age >65 years). No significant association between survival and anti-β1,6-glucan titers was instead found either in the whole population or in any category or sub-group of candidemic patients.

## Discussion

In this study, we report novel observations about antibody responses against antigenic constituents of an immunodominant polysaccharide-protein complex of the cell wall of *Candida* spp. in candidemic and non-candidemic, control patients. The saccharide antigens are the β1,3- and β1,6-glucan polymers which are known to be involved in many critical aspects of host-parasite relationship in candidiasis^29, 30^. The protein antigen is MP65, a putative endo-glucanase enzyme, usually found associated to β1,3-glucan, and previously identified as a major target of T cell responses to *C*. *albicans* ^27, 28^. Both β-glucans and MP65 have been shown to be expressed and abundantly secreted by yeast and hyphal cells of *C*.*albicans* in which they play an essential physiological role for cell wall growth and pathogenesis, including adherence to host cells and biofilm formation^28, 30^. Less is known regarding these antigens in other pathogenic *Candida* spp., where, however, they are present^31^ and possibly play similar roles as in *C*. *albicans*. Because of their strong involvement in the fungal biology and immune response, we considered of special relevance to assess the antibody response to these constituents in candidemic subjects. In fact, few studies have addressed antibody responses in candidemic subjects, mostly for diagnostic purposes^17–24^, and none of them specifically investigated the antibodies to the β-glucan complex of *Candida* cell wall. A report by Pitarch and coworkers describing a *C*. *albicans* immune serological proteome^23^ showed that candidemic subjects have serum antibodies against a high number of fungus proteins and their peptide sequences, most of which were poorly or not represented at all in non-candidemic subjects. Other authors have recently reported on the presence in candidemic subjects of dominant antibodies against proteins secreted during yeast to hypha transition of *C*. *albicans* and highlighted the importance of the glycosylated moieties of these proteins as target of antibody responses^19^. Overall, non-immunocompromised candidemic patients are evidently able to raise anti-*Candida* responses during the infection, some of which have also been assumed to be of diagnostic relevance^21, 22, 24, 25^. Here we report that candidemic patients, at the time of candidemia diagnosis, had significantly higher titers of anti-MP65 and anti-β1,6-glucan antibodies than non-candidemic subjects. The higher anti-MP65 titers of candidemic patients were not restricted to those infected by *C*. *albicans*, corroborating the notion that this important antigen is fully expressed also in other pathogenic species of *Candida* ^31^.

It is known that *C*. *albicans* colonization of mucosal surfaces, in particular the GI tract, and skin of most healthy subjects induces strong humoral and cellular responses^32^. The antibody levels against the β1,6-glucan moiety of the glucan/MP65 complex in non-candidemic, but likely *Candida*-colonized subjects, were in the same range of those previously reported in normal healthy subjects^29^. Hence, the elevated titers of these antibodies in candidemic patients (reasonably including those againstMP65) are most likely due to a booster of anti-*Candid*a immune response to fungus invasion, that is expected in a generally non-immunocompromised patient population. Actually, the above booster was seen in all categories of candidemic patients irrespective of their age, sex, performance, antifungal treatment and co-morbidity (with the possible exception of diabetic patients; see below) and occurred against both the polysaccharide and, particularly high, against the protein antigen.

Despite the existence of protective anti-*Candida* antibodies, amply documented in experimental studies^33–35^, very little is known about the existence and impact of such antibodies in clinical setting. The large majority of the studies done so far have addressed the diagnostic, rather than the prognostic, role of anti-*Candida* antibodies. Here we examined whether there was any association between levels of anti-β1,6-glucan or anti-MP65 antibodies and disease outcome in our candidemia setting. Overall, our data show a statistically significant association between survival and antibody responses against the MP65, but not against the β1,6-glucan, antigen in the whole candidemia population, with the expected particular strength when anti- MP65 antibody titers and survival were correlated in the categories of candidemic subjects where most of the mortality is observed (older age, diabetics, septic shock patients). Notably, the anti-MP65 titers of the diabetic, non-survivors were very close to those of non-candidemic subjects, suggesting for the inability of these patients to boost a substantial anti-*Candida* response following infection.

These data do not allow to conclude that MP65 is a protective antigen in candidemia, since it could only be an indirect marker of immune response, even of non-antibody type, against MP65 itself and/or other antigens. Bloodstream infection and sepsis is expected to cause some immune dysregulation at least in those categories of candidemic subjects heavily invaded by the fungus. Disorders in T cell and pro-inflammatory cytokines have indeed been reported that could, in some subjects, interfere with the booster of antibody responses^17, 36^. In particular, patients with *Candida* sepsis have been reported to show a suppressive immune-phenotype including T cell exhaustion and decreased expression of co-stimulatory molecules in CD4^+^ T cells^36^. It is therefore conceivable that in some candidemic subjects the CD4^+^ T cells are unable to express their full helper potential for antibody production. Our observation that the anti-MP65 but not the anti-β1,6-glucan antibodies had lower titers in non-survivors is in accord with the above hypothesis, being MP65 and β1,6-glucan, respectively, a T-helper-dependent and -independent antigen. Whichever the interpretation, our data suggest that the subjects unable to mount a more intense or more specific anti-*Candida* immunity following infection could have a worse outcome.

Our study has the well-known limitations of a retrospective one, and reporting only from a single, though large, clinical center. Some categories of subjects with high proneness to candidemia and its associated mortality (e.g. diabetics) were scarcely represented among both patients and controls. In addition, the non-candidemic, control patients were not perfectly matched for some morbidities to the candidemic patients, although belonging to the same at risk categories and largely sharing the same predisposing factor Overall, our suggestion that the capacity to boost anti-Candida immunity following infection might have an impact on candidemia survival, though being statistically supported, remains highly speculative and, as such, should be taken cautiously. Nonetheless, our data invite to consider further prospective and possibly multicenter studies to assess whether antibody measurements against properly selected fungal antigens could help identify candidemic subjects with differential survival potential. These studies could suggest ways of harnessing immune responses to the infectious fungi in some categories of candidemic subjects by appropriate immune-stimulation (including vaccination) in order to improve the prognosis in a clinical setting characterized by particularly high mortality.

## Methods

### Study population and design

We included in the study patients with culture-proven candidemia who were hospitalized during the period from January 2012 to February 2016 at the A. Gemelli Hospital of Rome, Italy, which is an academic tertiary care center with 1,400 beds and ~50,000 hospital admissions per year. Patients were identified by electronically querying the clinical microbiology laboratory database, and were included only if complete data series could be retrieved from their medical charts and from laboratory databases, including demographics (age, sex), comorbid conditions (diabetes mellitus, chronic obstructive pulmonary disease, chronic renal failure, solid organ cancer, hematologic malignancy, human immunodeficiency virus [HIV] infection), insertion of a central venous or urinary catheter, use of immunosuppressive agents, surgery, exposure to antibiotics within 30 days of the onset of candidemia, risk days (i.e., number of hospital days from admission to the date of the first positive blood culture, antifungal therapy, and hospital mortality [the last defined as death within 30 days of the first documented candidemia episode]). The local institutional review committee approved the study, and informed consent was waived because of the observational, retrospective nature of the investigation. All patient samples and data records were rendered anonymous before performing any experimental or statistical analyses.

Diagnosis of candidemia was made on the basis of ≥1 blood cultures growing *Candida* species and on the presence of signs and symptoms of infection. Only the first episode of candidemia was reported for patients with recurrent or subsequent episodes of infection. In addition, only patients with β1,3-glucanemia assay results (see below) were included.

Controls were age- and sex-matched patients who were admitted to the hospital within the same time interval as the candidemic ones, and were considered at risk for candidemia but with *Candida*-negative blood-culture and negative β-glucanemia. Only candidemic and non-candidemic patients whose serum was available in suitable quantity and quality for performing immunoassays were considered.

### Microbiological assays

Blood cultures were obtained as part of normal clinical practice and processed using a Bactec (BD Diagnostic Systems, Sparks, MD) or BacT/Alert (bioMérieux, Marcy l’Etoile, France) system. After sub-culturing on Difco *Candida* bromocresol green (BCG) agar medium, yeast isolates were identified to the species level by matrix-assisted laser desorption ionization–time of flight (MALDI-TOF) mass spectrometry^37^. Sera from blood specimens of candidemic and non-candidemic controls, as described in the previous section, were tested for β1,3-D-glucan (BDG; Fungitell; Associates of Cape Cod Inc., Falmouth¸ MA) as previously described^38^. The concentration of BDG in each sample was automatically measured within a range from 31.25 to 500 pg/ml. Using the BDG cutoffs proposed by the manufacturer, we considered a BDG to be positive if the value was ≥80 pg/ml and negative if the value was <80 pg/ml.

### Immunological assays

Laminarin (essentially a beta-1,3-linked glucan with occasional beta-1,6-linked side chains of a single glucose residue) and pustulan (a linear, beta-1,6-linked glucan) were used as model antigens representing β1,3- and β1,6- *Candida* glucans, respectively^29^. They were purchased from Sigma Chem. Co. (St. Louis, MO) and Calbiochem (La Jolla, CA), respectively. Full length, poly-histidine tagged, recombinant MP65 protein was produced in *E*.*coli* by GenScript (Piscataway, NJ) and purified to homogeneity by nickel-affinity chromatography.

Sera of candidemic patients and non-candidemic controls, were extracted from the serum bank of the A. Gemelli Hospital microbiology laboratory. A single serum sample was analyzed for each subject under study. For candidemic patients, serum samples were those corresponding to laboratory diagnosis of the first candidemia episode (i.e., obtained from the same blood specimen of the first *Candida*-positive blood culture and found positive for presence of circulating β1,3-glucan). All sera were heated at 56 °C for 30 min and stored individually at −80 °C.

Immunoglobulin G (IgG) titers against the β-glucans and MP65 antigens were determined by ELISA, essentially as already described^29^. Briefly, polystyrene microtiter plates (MaxiSorp; NUNC, Roskilde, Denmark) coated with the antigens (50 μg/ml polysaccharide or 0.5 μg/ml MP65 in 0.05 M carbonate buffer, pH 9.6, 100 μl/well, overnight at + 4 C) and blocked 2 h at 37 °C with Blocker ^TM^ Casein in PBS (Thermo Scientific, Rockford, IL, USA), were reacted with twofold dilutions of the human sera in blocking solution, followed by alkaline phosphatase-conjugated, gamma chain-specific, goat anti-human IgG (Sigma). Plates were developed 30 min with p-nitrophenyl phosphate disodium (Sigma) as the enzyme substrate and read for absorbance at 405 nm. Readings from negative control wells (wells without antigen) were subtracted from all absorbance values. Antibody titers were defined as the reciprocal of the highest dilution of sera that gave an optical density at least twice that of the correspondent negative control.

For western blot analyses, the MP65 protein preparation (2.5 μg/lane) was separated by SDS-PAGE in 5 to 15% polyacrylamide slab gels (Bio-Rad, Hercules, CA) and transferred onto nitrocellulose (0.2-mm pore size), as previously described^27^. After blocking of the nitrocellulose sheets for 2 h at room temperature with Blocker ^TM^ Casein in PBS (Thermo Scientific), the transferred protein was reacted for 1 h at 37 °C with control or patient sera (diluted 1:200 in Blocker ^TM^ Casein in PBS) or with a murine, anti-MP65 monoclonal IgG (7H6^27^, 5 μg/ml in the same diluent of human sera). Sheets were extensively washed with PBS and incubated with alkaline phosphatase-conjugated, anti-human or anti-mouse IgG antibodies, as appropriate. Reactive bands were developed with the nitroblue tetrazolium chloride/5-bromo-4-chloro-3-indolyl phosphate reagent (Sigma).

### Statistics

Categorical variables were compared using the Fisher’s exact test or the Chi-square test. Comparison of continuous data among groups were performed with the Mann-Whitney *U*-test, confirmed by the Student’s t test, whereas one-way ANOVA or Kruskal-Wallis test followed by Bonferroni’s or Dunn’s post-tests, respectively, were used for multiple comparisons. Receiver-operator characteristics (ROC) curves were constructed to evaluate the potential of analyzed antibodies for an accurate discrimination between candidemic and non-candidemic patients. Spearman’s rank correlation coefficient was calculated to assess the relationships between the different variables in patients. A p value < 0.05 was accepted as statistically significant. All tests were performed with the GraphPad Prism software, version 4.00 (GraphPad, San Diego CA). Relationship between survival and logarithm to base 10 of anti-MP65 and anti-β1,6-glucan antibody titers in all candidemic patients and in patient subgroups was assessed by logistic regression analyses by the SPSS v19 software.

## Electronic supplementary material

Supplementary Information
