# Supplementary material for: Antibodies against a β-glucan-protein complex of Candida albicans and its potential as indicator of protective immunity in candidemic patients
Source: Sci Rep. 2017 Jun 2;7:2722. doi: 10.1038/s41598-017-02977-6 (PMC5457410; doi:10.1038/s41598-017-02977-6)
Supplement: Supplementary file 1 — Supplementary Information [file 41598_2017_2977_MOESM1_ESM.pdf]

## SUPPLEMENTARY INFORMATION

# Antibodies against a β-glucan-protein complex of Candida albicans and its potential as indicator of protective immunity in candidemic patients

**Antonella Torosantucci, Mario Tumbarello, Carla Bromuro, Paola Chiani, Brunella Posteraro, Maurizio Sanguinetti, Roberto Cauda and Antonio Cassone ***

**Supplemental Table S1.** Clinical and demographic data of controls

| | N/Total | % |
|---|---|---|
| Age: 69 (median), 18-89 (range) | - | - |
| Male | 37/69 | 53.6 |
| Female | 32/69 | 46.4 |
| | | |
| Central venous catheter | 25/69 | 36.2 |
| Previous surgery | 30/69 | 43.5 |
| Hematomalignancy | 14/69 | 20.3 |
| HIV | 1/69 | 1.4 |
| Immunosuppressant | 7/69 | 10.1 |
| Steroids | 2/71 | 2.9 |
| Solid tumor | 14/69 | 20.3 |
| Chronic obstructive pulmonary disease | 2/69 | 2.9 |
| Chronic renal failure | 3/69 | 4.3 |
| Diabetes mellitus | 6/69 | 8.7 |
| Previous antibiotic treatment (30 days) | 39/69 | 56.5 |

**Supplemental Figure S1.** Anti-MP65 and anti-β1,6-glucan antibody titers in candidemic patients and in non candidemic controls: ROC curves

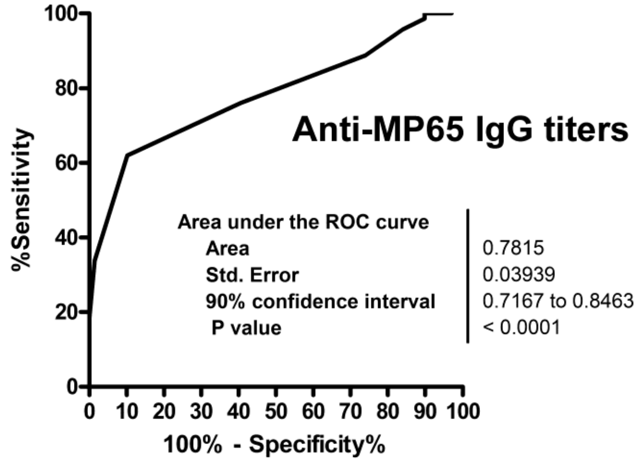


| Cutoff | Sensitivity% | 95% CI | Specificity% | 95% CI | Likelihood ratio |
|---|---|---|---|---|---|
| > 600.0 | 88.73 | 79.00% to 95.01% | 26.09 | 16.25% to 38.06% | 1.20 |
| > 1200 | 76.06 | 64.46% to 85.39% | 59.42 | 46.92% to 71.09% | 1.87 |
| > 2400 | 61.97 | 49.67% to 73.24% | 89.86 | 80.21% to 95.82% | 6.11 |
| > 4800 | 33.80 | 23.00% to 46.01% | 98.55 | 92.19% to 99.96% | 23.32 |

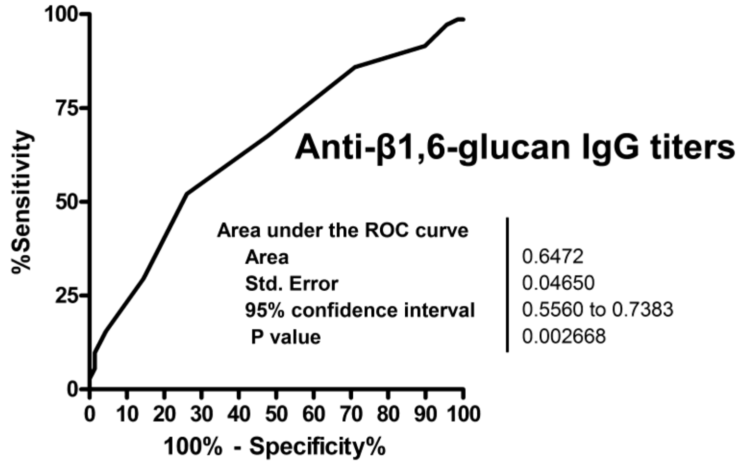


| Cutoff | Sensitivity% | 95% CI | Specificity% | 95% CI | Likelihood ratio |
|---|---|---|---|---|---|
| > 2400 | 85.92 | 75.62% to 93.03% | 28.99 | 18.69% to 41.16% | 1.21 |
| > 4800 | 67.61 | 55.45% to 78.24% | 52.17 | 39.80% to 64.36% | 1.41 |
| > 9600 | 52.11 | 39.92% to 64.12% | 73.91 | 61.94% to 83.75% | 2.00 |
| > 19200 | 29.58 | 19.33% to 41.59% | 85.51 | 74.96% to 92.83% | 2.04 |

**Supplemental Figure S2.** Reactivity of sera from candidemic or non-candidemic patients with *C.albicans* MP65: western blot analysis.

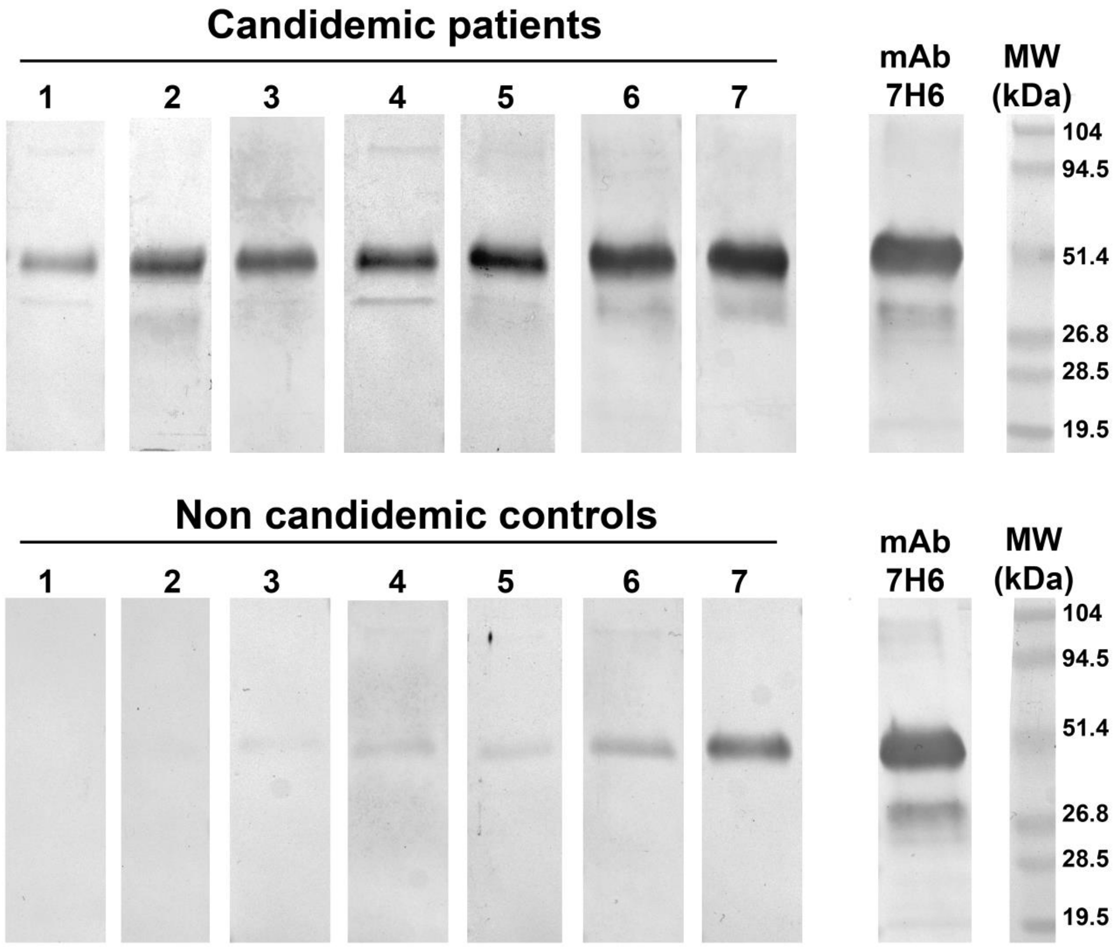


The figure shows a comparison of the reactivity profiles generated by sera of different candidemic or non-candidemic patients and by an MP65-specific, murine monoclonal antibody (mAb 7H6). Anti-MP65, ELISA titers of analyzed subjects were as follows: candidemic patients 1 and 2, 1:1600; 3, 4 and 5, 1:3200; 6 and 7, 1: 12800; non candidemic controls 1, 2 and 3, 1:200; 4 and 5, 1:800; 6, 1:1600; 7, 1:6400.
